# Supplementary material for: Ecological memory of recurrent drought modifies soil processes via changes in soil microbial community
Source: Nat Commun. 2021 Sep 6;12:5308. doi: 10.1038/s41467-021-25675-4 (PMC8421443; doi:10.1038/s41467-021-25675-4)
Supplement: Supplementary file 1 — Supplementary information. [file 41467_2021_25675_MOESM1_ESM.pdf]

Supplementary figures

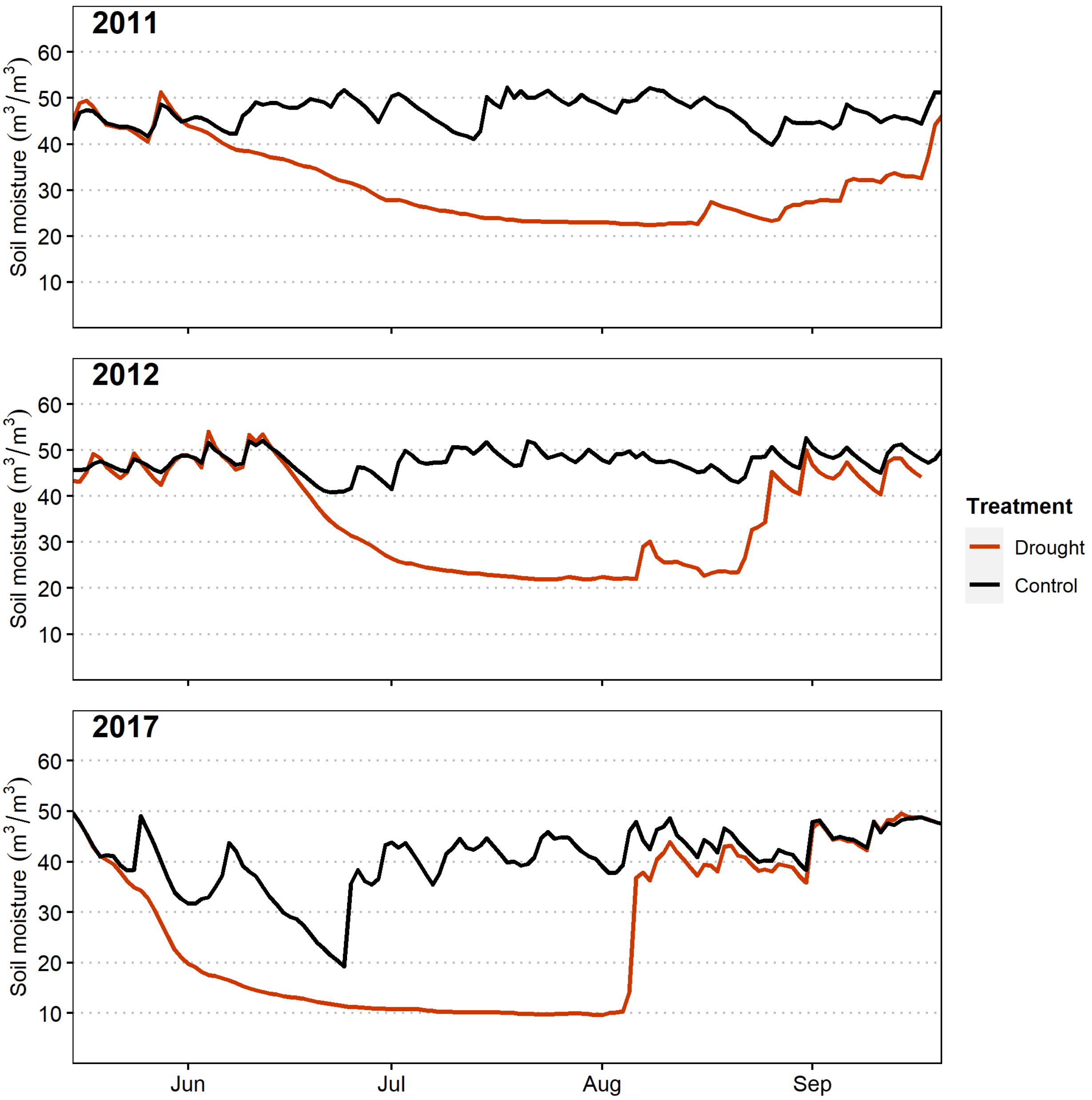

Supplementary Figure 1. Soil moisture and temperature during the experimental years of sampling (2011, 2012 and 2017) for a representative control plot and a drought treatment, measured at 10 cm depth.

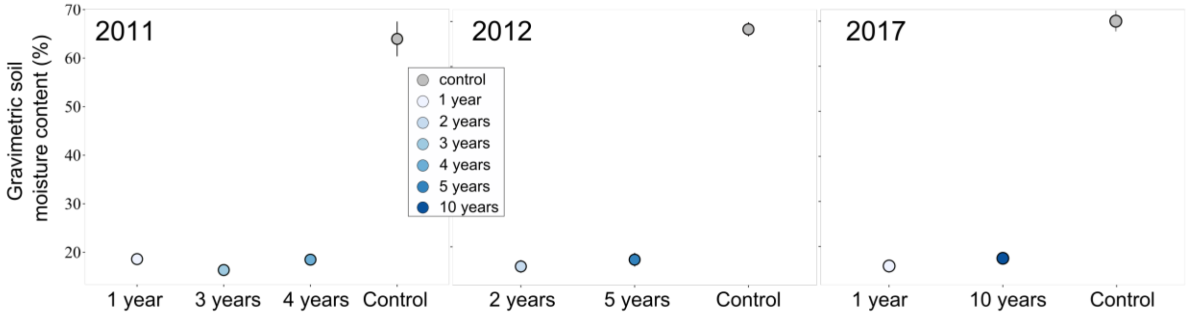

Supplementary Figure 2. Soil moisture measured gravimetrically after soil sampling, in the three investigated years (2011, 2012 and 2017) for all treatments. Points represent mean and bars represents standard error of the mean (n=4, representing biologically independent samples).

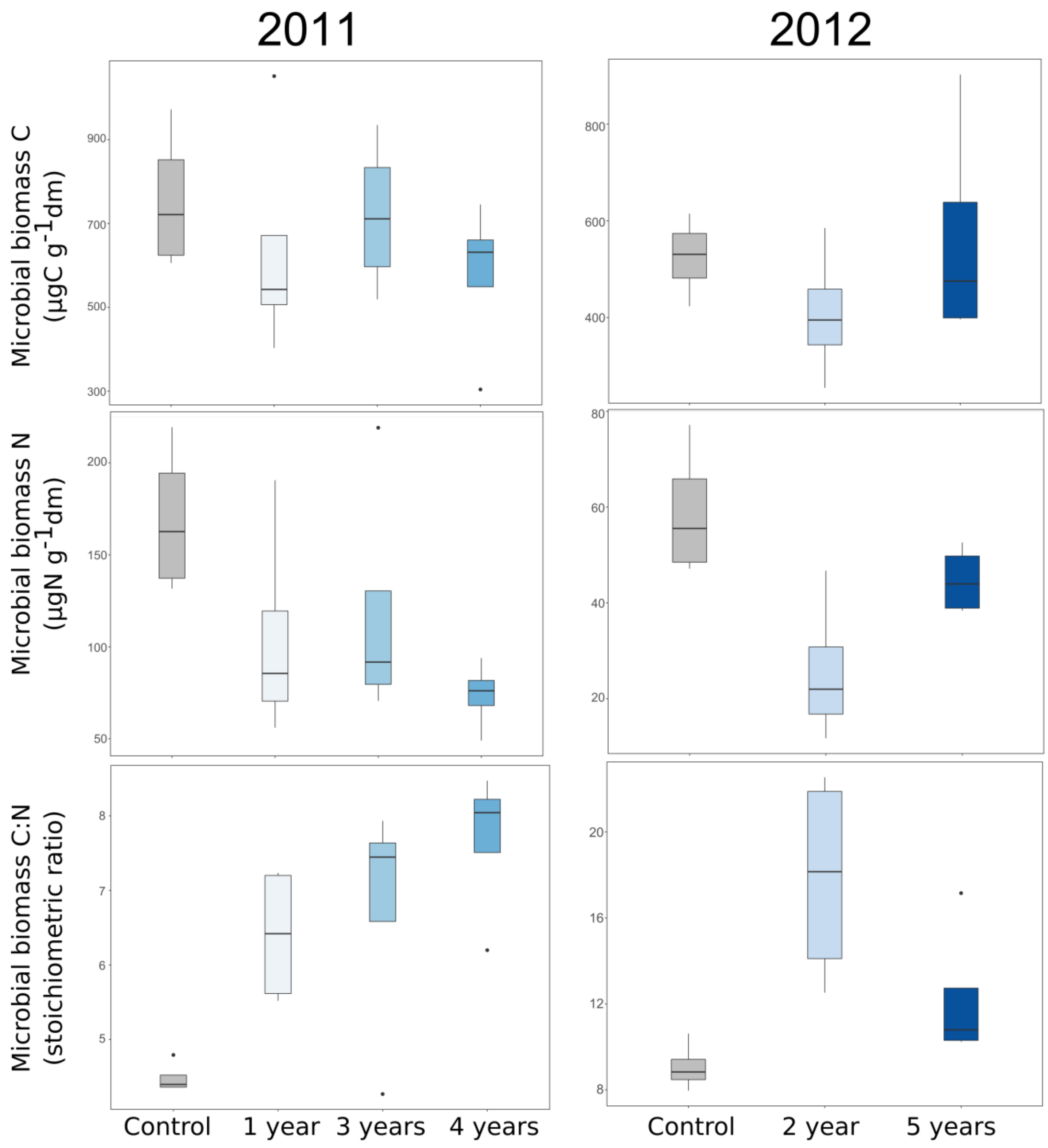

Supplementary Figure 3. Microbial stoichiometric values for the 2011 and 2012 campaigns. Top graphs show microbial biomass carbon, where no significant effects of drought were found (n=4). Middle graphs show microbial biomass nitrogen, where drought decreased N in microbial biomass (2011: p=0.075, 2012: p=0.014, n=4). Bottom graphs show the stoichiometric ratios of C to N, where drought increased this ratio (2011: p=0.009; 2012: p=0.019, n=4). One-way ANOVA was used to test the main effects of treatments within each sampling campaign. Box center line represents median, box limits the upper and lower quartiles, whiskers the 1.5x interquartile range, while separated points represents outliers.

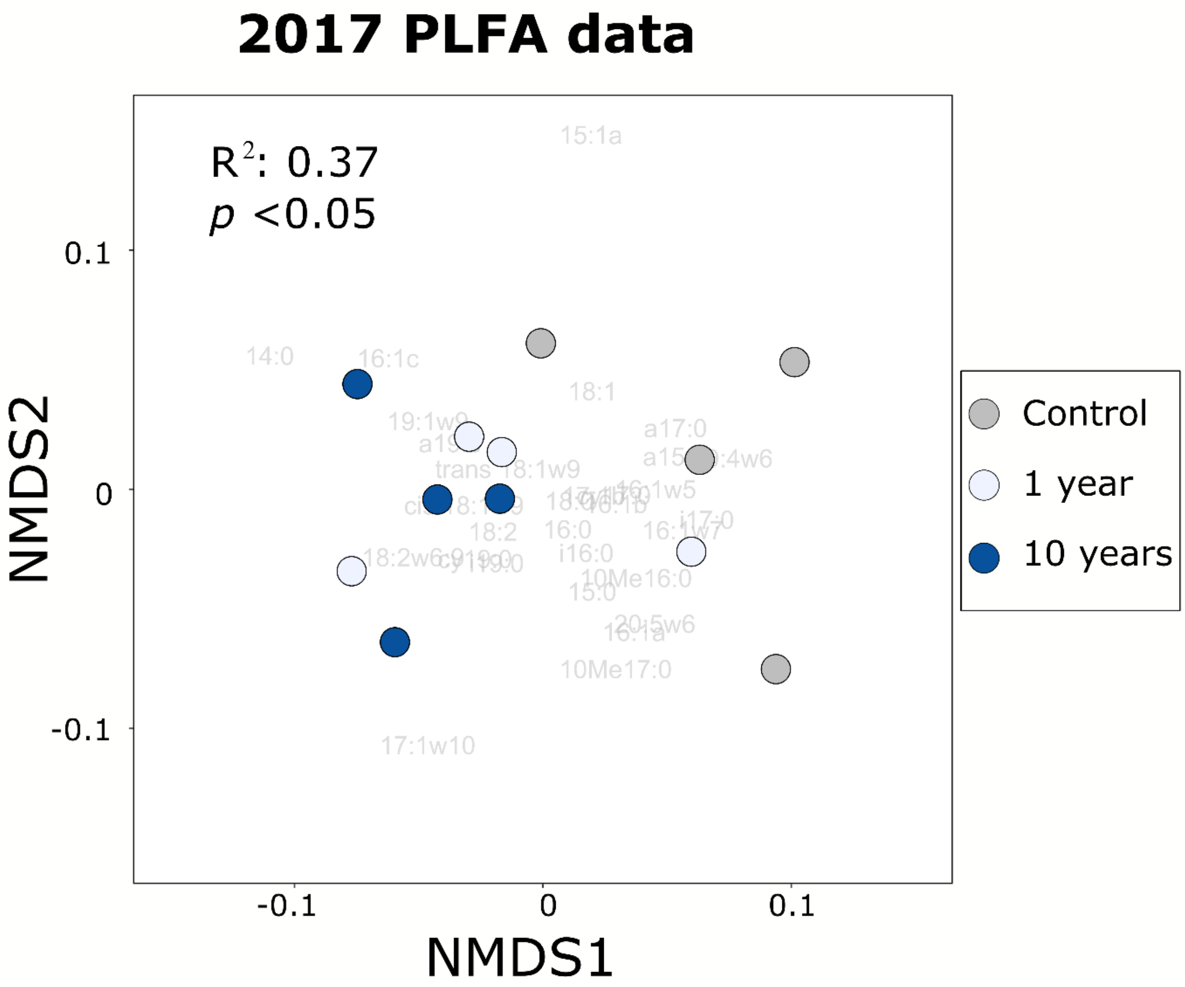

Supplementary Figure 4. Graph represents NMDS of the PLFA biomarkers in the 2017 campaign and show a significant effect of drought (PERMANOVA: R^2=0.37 and p=0.017, n=4).

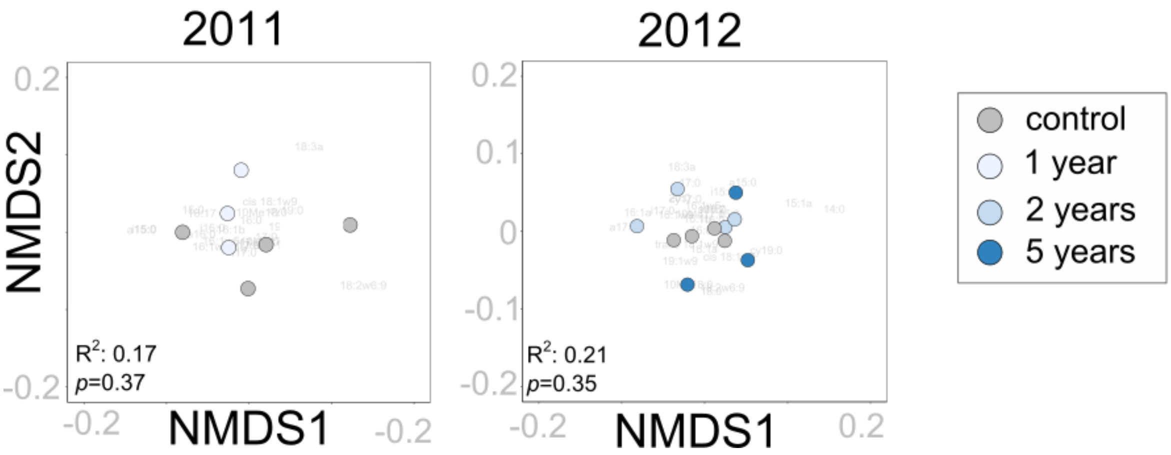

Supplementary Figure 5. Effects of drought on the soil microbial community composition measured by PLFAs. Graphs represent the NMDS of relative abundance of PLFA biomarkers for the year 2011 and 2012. We found no significant separation between treatments (PERMANOVA 2011: R^2=0.37 and p=0.17, n=3; PERMAOVA 2012: R^2=0.35 and p=0.21, n=4 except 5 years treatment where n=3).

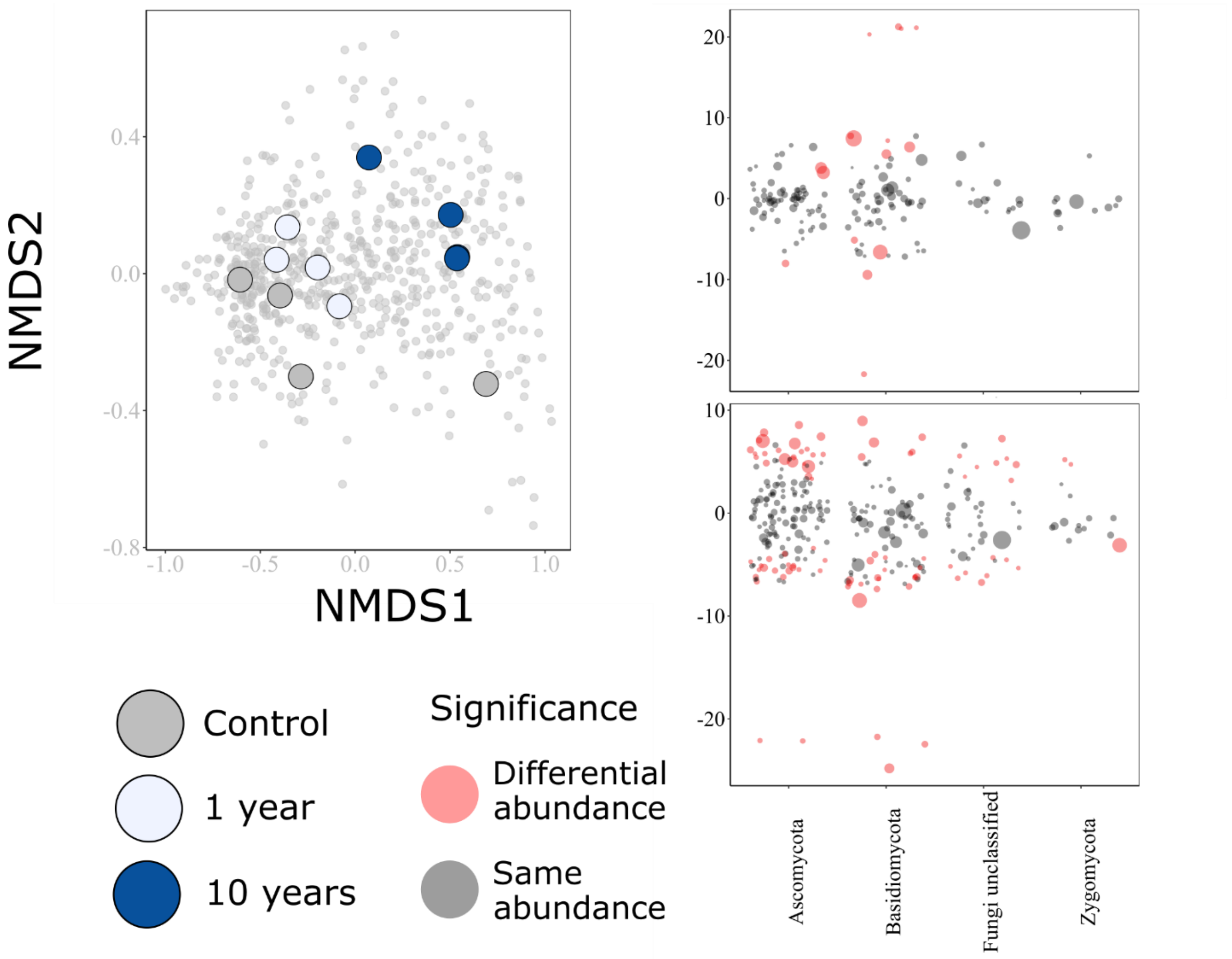

Supplementary Figure 6. Drought effects on microbial community composition assessed by amplicon sequencing (ITS2, fungi). NMDS plot shows an effect of drought (PERMAOVA, p=0.006, n=4) and a separation of 10 years treatment from control and 1 year. The right graphs show results from the differential abundance analysis for control vs 1 year (top right graph) and control vs 10 years (right bottom row). Circles in red represent taxa that had a significant differential abundance compared to the control.

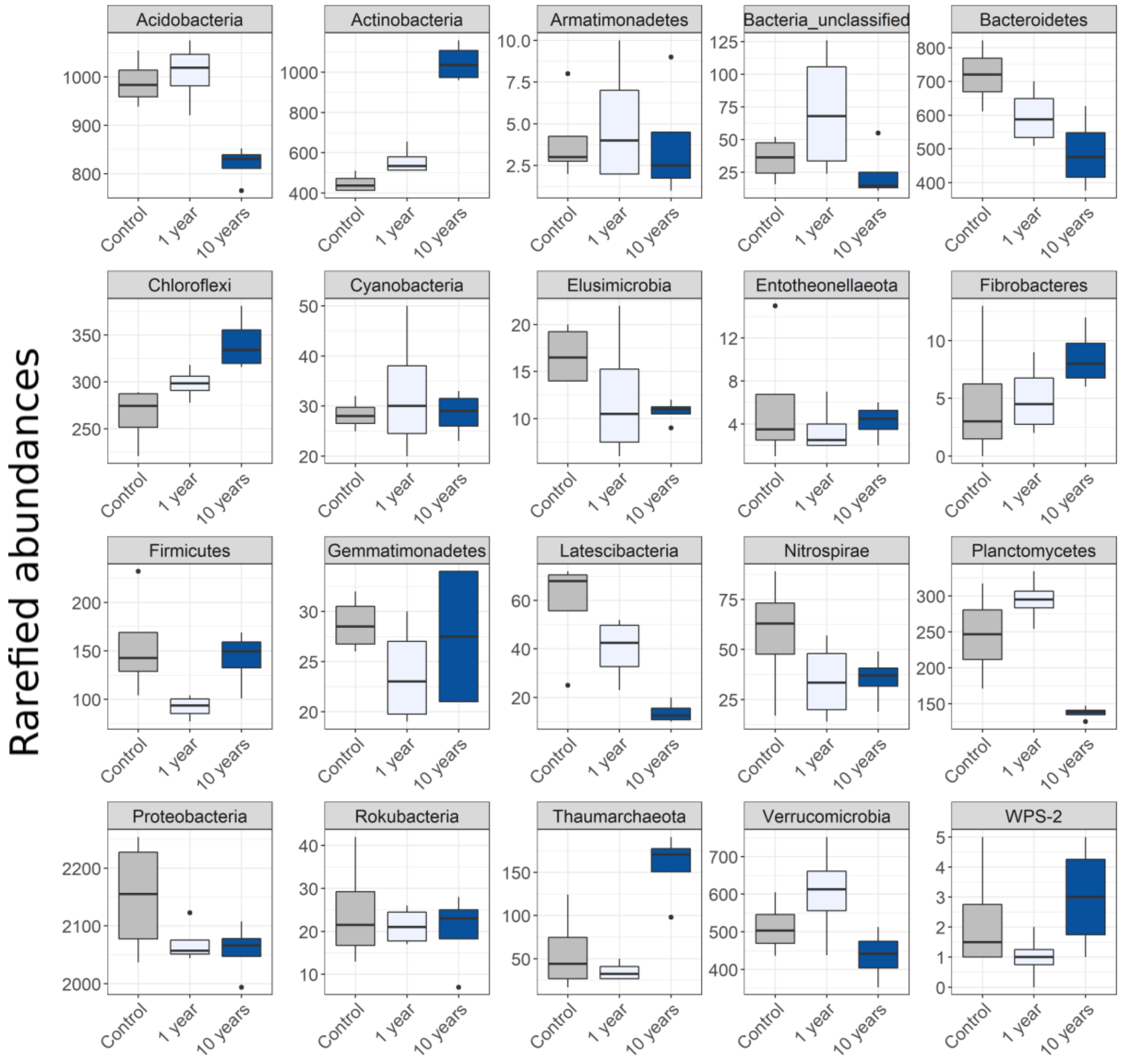

Supplementary Figure 7. Boxplots representing rarefied abundances (rarefied to 5809 representing the minimum sample size) of Phyla found with the 16S primer. Color indicates soil treatment (Control = grey, 1 year = light blue, 10 years = blue). Box center line represents median, box limits the upper and lower quartiles, whiskers the 1.5x interquartile range, while separated points represents outliers.

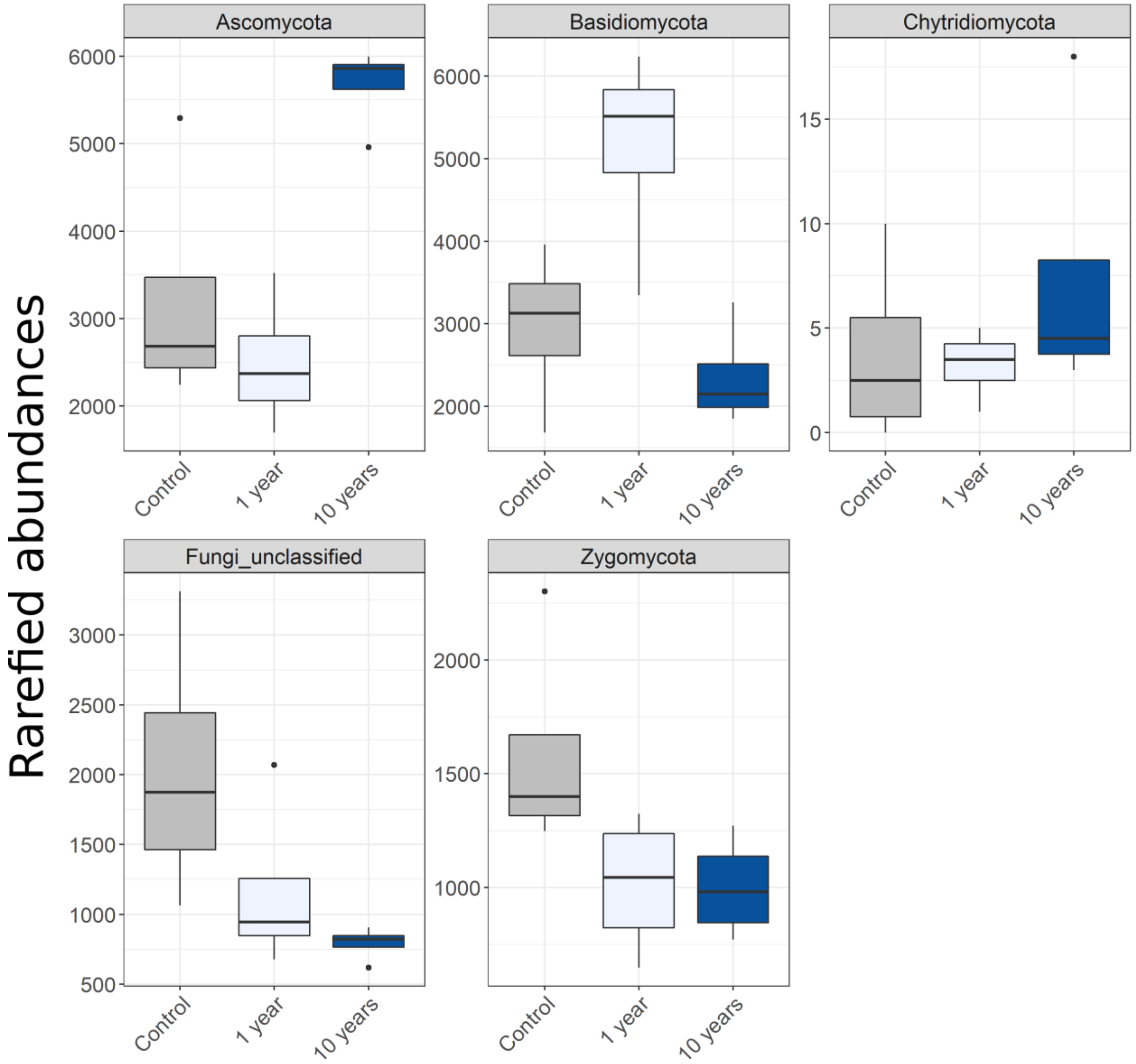

Supplementary Figure 8. Boxplots representing rarefied abundances (rarefied to 9820 representing the minimum sample size) of Phyla found with the ITS1 primer. Color indicates soil treatment (Control = grey, 1 year = light blue, 10 years = blue). Box center line represents median, box limits the upper and lower quartiles, whiskers the 1.5x interquartile range, while separated points represents outliers.

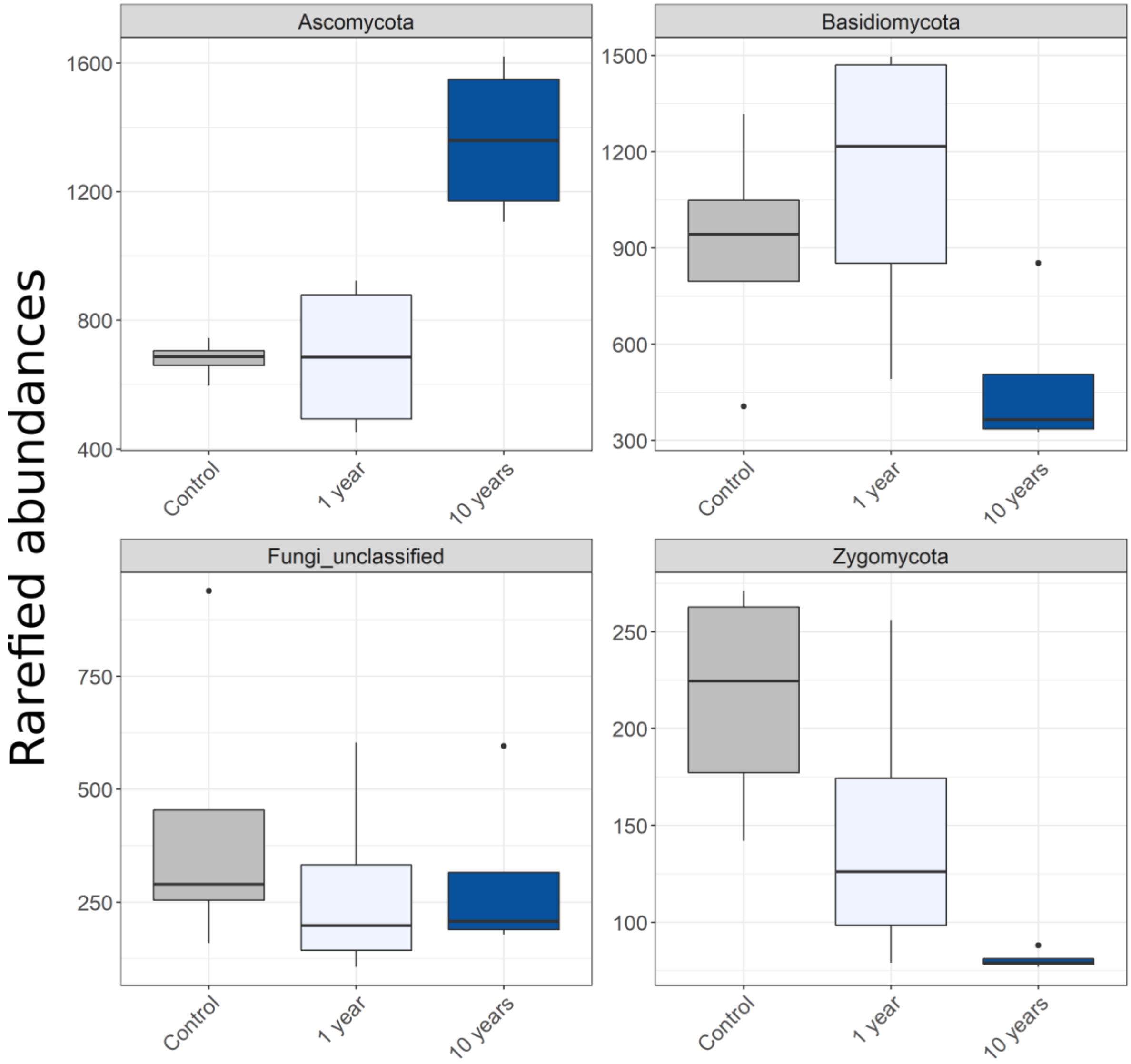

Supplementary Figure 9. Boxplots representing rarefied abundances (rarefied to 2216 representing the minimum sample size) of Phyla found with the ITS2 primer. Color indicates soil treatment (Control = grey, 1 year = light blue, 10 years = blue). Box center line represents median, box limits the upper and lower quartiles, whiskers the 1.5x interquartile range, while separated points represents outliers.

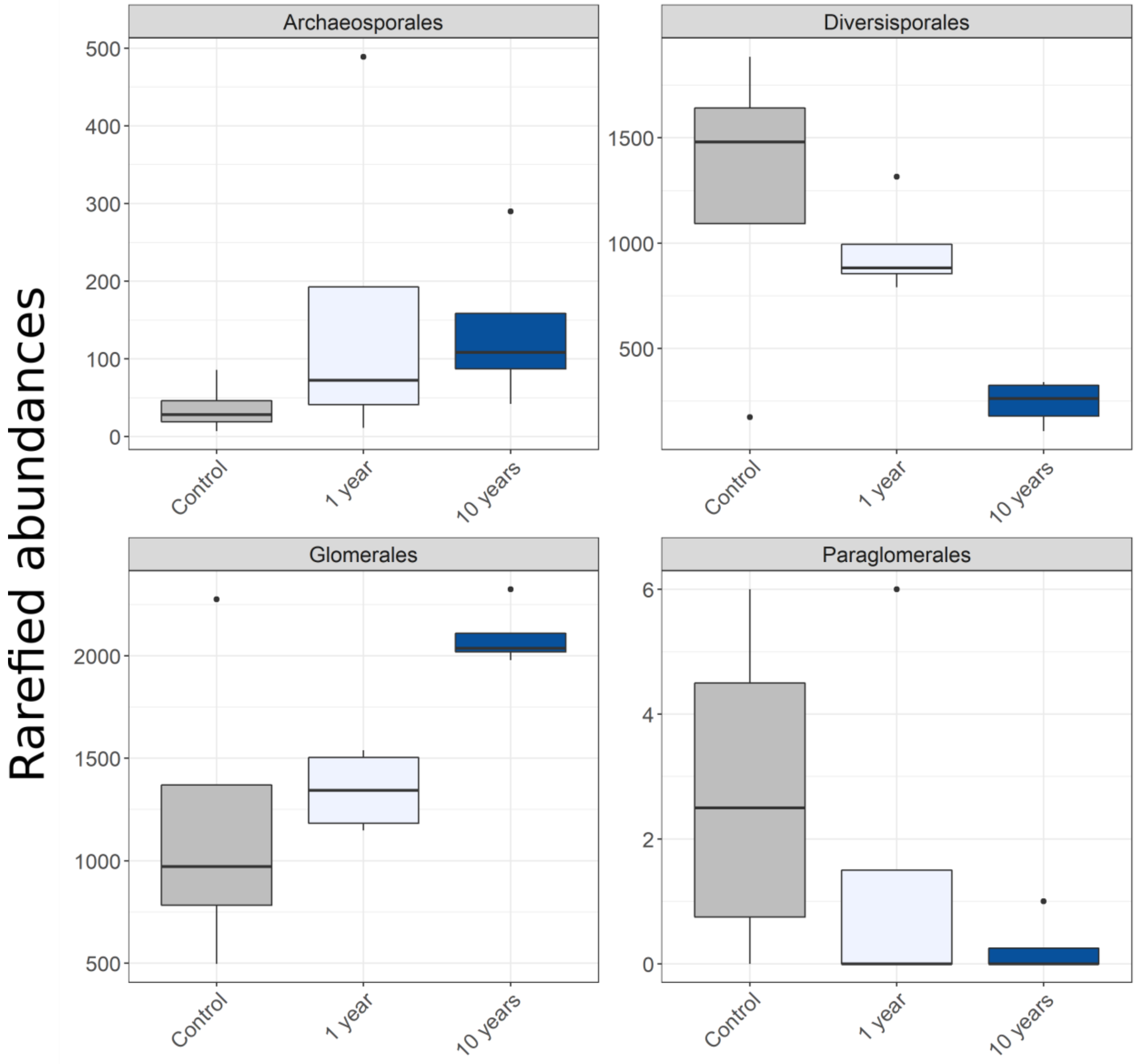

Supplementary Figure 10. Boxplots representing rarefied abundances (rarefied to 2474 representing the minimum sample size) of Orders found with the AMF primer. Color indicates soil treatment (Control = grey, 1 year = light blue, 10 years = blue). Box center line represents median, box limits the upper and lower quartiles, whiskers the 1.5x interquartile range, while separated points represents outliers.

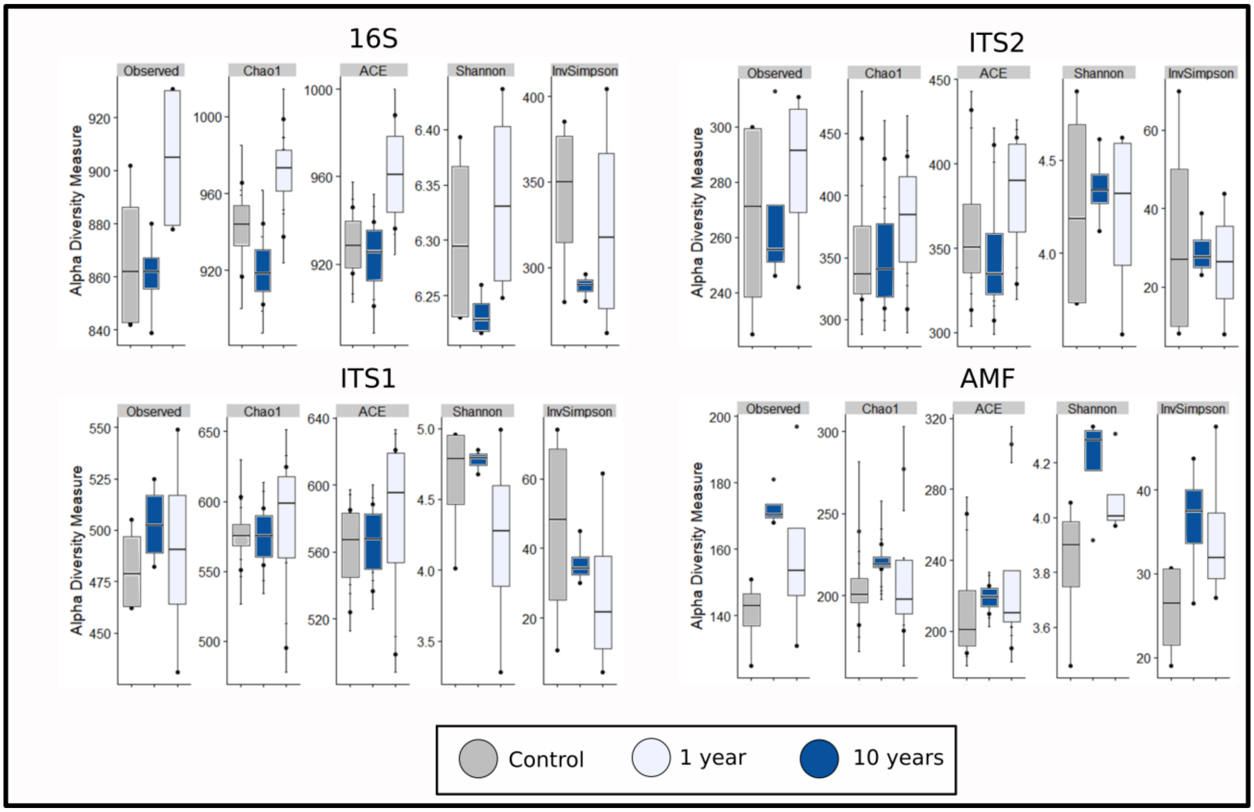

Supplementary Figure 11. α-diversity indices generated by the package phyloseq. Box center line represents median, box limits the upper and lower quartiles, whiskers the 1.5x interquartile range, while separated points represents outliers (n=4).

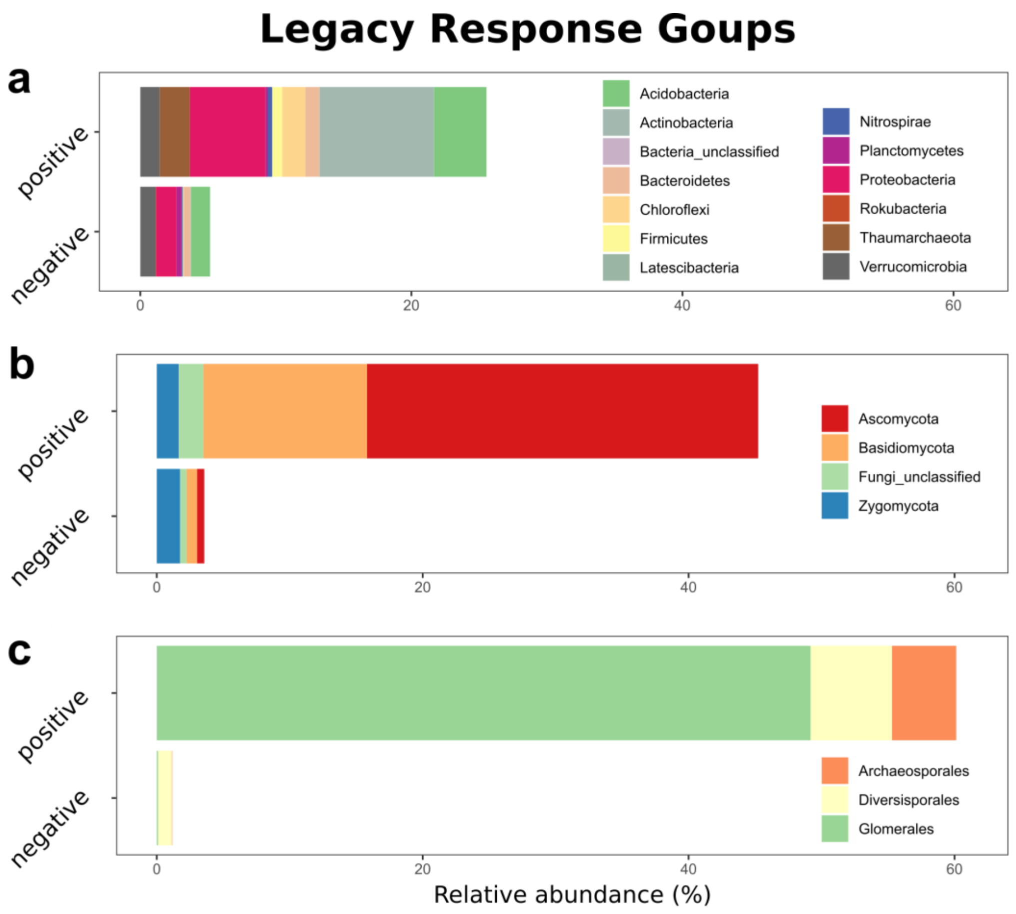

Supplementary Figure 12. The relative abundance of the Legacy Response Groups (LRGs) for a) 16S, b) ITS and c) AMF dataset. Taxonomic affiliations of each group are shown within each graph (Phylum for a and b and Order for c). "Positive" indicates group that show enrichment in the 10-years treatment vs 1-year treatment, while "negative" indicates a decrease (following deseq analysis).

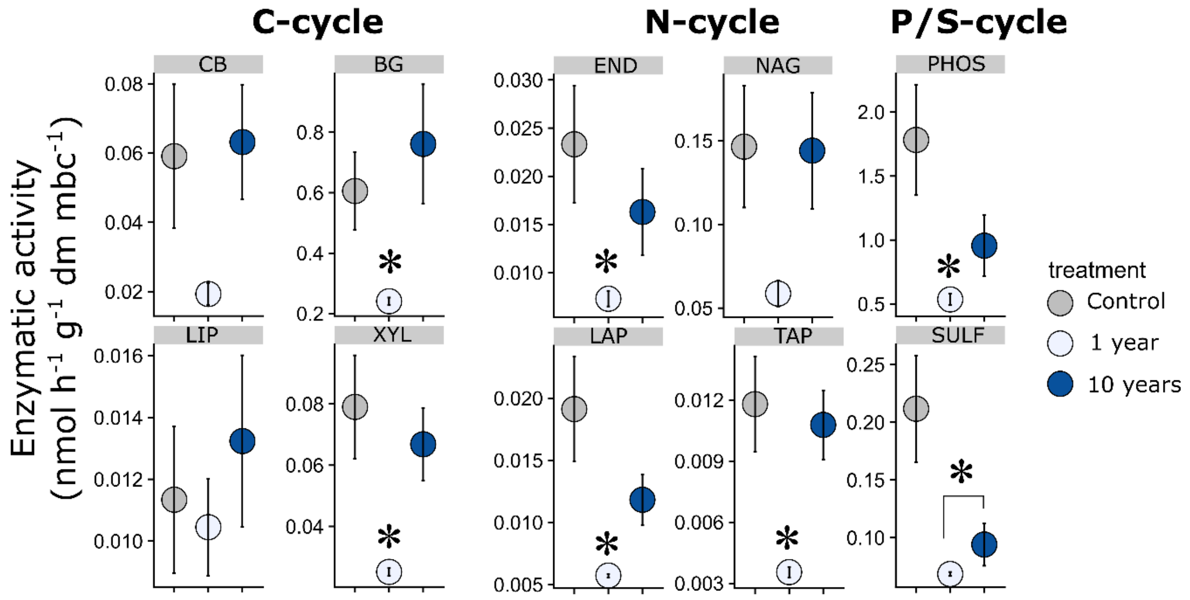

Supplementary Figure 13. Enzymatic activity normalized by microbial biomass carbon. Enzyme abbreviations are explained in Supplementary Table 3. Significant effects of drought were found for enzymes BG (P=0.021); XYL (P=0.006); END (P=0.044); LAP (P=0.006); TAP (P=0.001); PHOS(P=0.029) and SULF (P= 0.008). Asterisks indicate treatments being significantly different from control obtained by two-sided Tukey's HSD test at p<0.05. Points represent mean values and error bars the standard deviation (n=4).

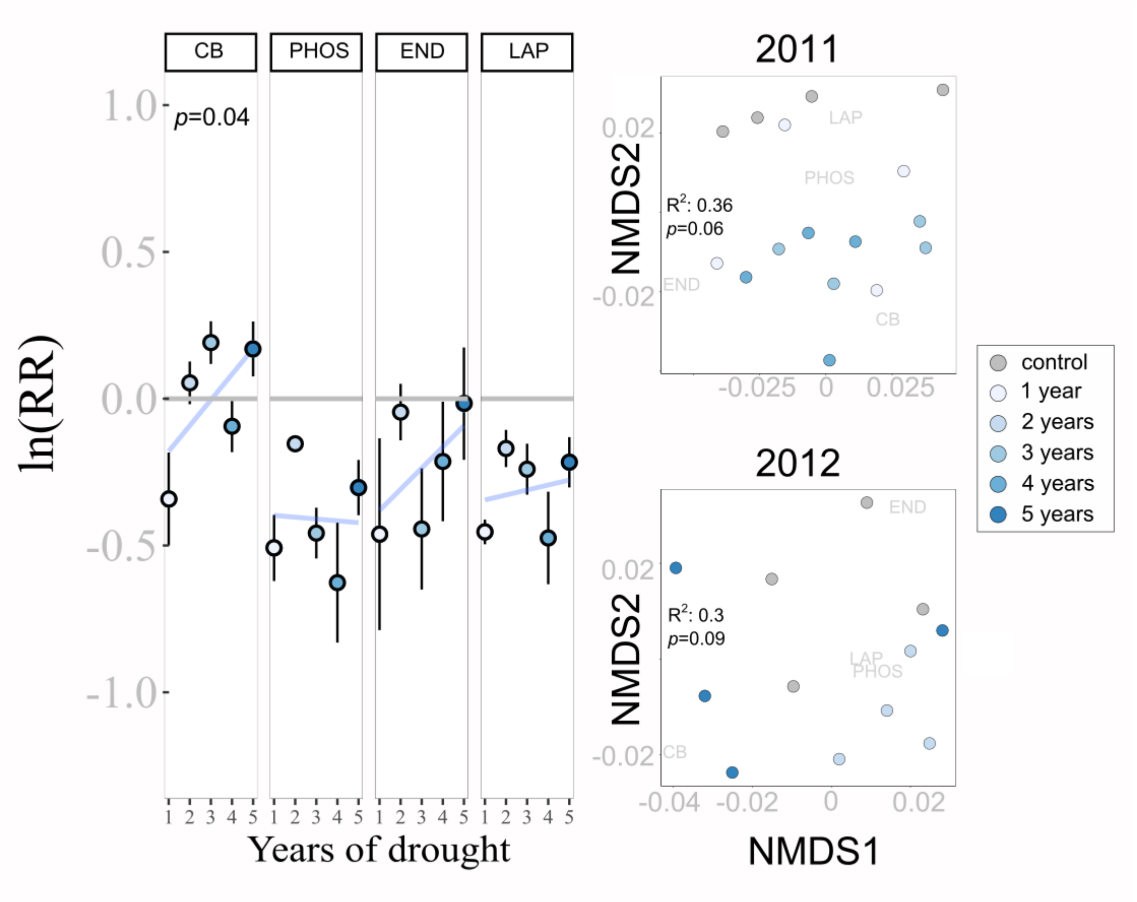

Supplementary Figure 14. Effects of drought on the potential enzymatic activity. Left graphs represent linear relationships between calculated response ratios and the number of previous years of drought for the measured enzymatic activities. Significant p values are reported. Right graphs represent the NMDS of potential enzymatic activities for the years 2011 and 2012 and statistical analysis obtained by PERMANOVA (n=4).

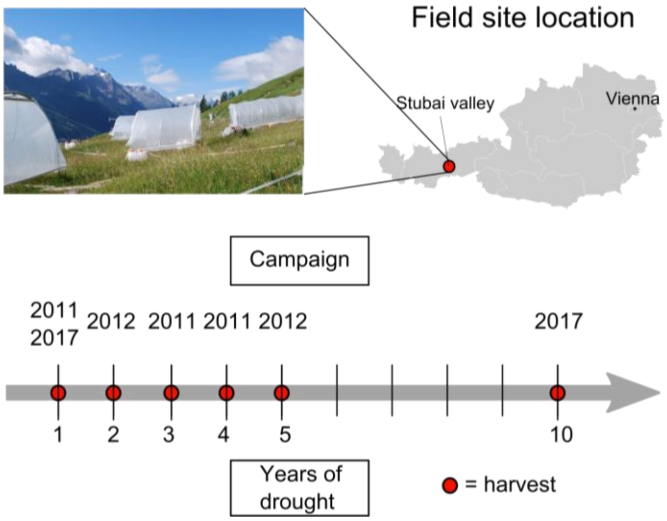

Supplementary Figure 15. Location and summary of the experimental set up. The left panel represents the location and a photograph of the experimental set up. Drought was simulated by using rain-out shelters during the summer season (from end of May to the beginning of August). The right panel shows a schematic representation of the sampling campaigns: three campaigns were carried out in 2011, 2012 and 2017. Control plots (no drought) were sampled at all campaigns. In 2011 samples from plots subjected to 1, 3, and 4 years of drought were also collected. In 2012 samples from plots subjected to 2 and 5 years of drought were collected. And in 2017 samples from plots subjected to 1 and 10 years of drought were collected.

Supplementary tables:

Supplementary Table 1. Plant and soil variables' response to drought (control vs 1year vs 10 years). Different variables were measured, including plant and soil-related parameter. Numbers indicate mean followed by ± standard error in brackets. ANOVA p-value are reported in bold, when p-value are below the 0.05 threshold. Letters indicate results from two-sided Tukey's HSD test.

Variable	Unit	Control	1 year drought	10 years drought	ANOVA p-values
Soil C	%	6.8 (± 0.25) a	6.6 (± 0.76) a	7.9 (± 0.51) a	0.227
Soil N	%	0.66 (± 0.03) a	0.61 (± 0.06) a	0.74 (± 0.04) a	0.228
Soil P	%	0.13 (± 0.01) a	0.10 (± 0.01) a	0.11 (± 0.01) a	0.112
EOC	µg C/g-DW	155.1 ±(22.7) a	355.1 ± (35.1)b	434.1 ±(67.1) b	**0.005**
EON	µg N/g-DW	32.07 ±(2.42) a	49.92 ± (7.13) a	51.78 ± (5.34) a	0.052
NH4+	µg NH4-N/g-DW	2.96 ±(0.81) b	13.85 ± (3.63) a	8.98 ±(1.75) ab	**0.031**
NO3-	µg NO3-N/g-DW	3.48 ±(2.28) a	1.08 ±(0.54) a	2.43 ±(0.85) a	0.523
EOP	µg P/g-DW	6.03 ±(2.93) a	7.13 ±(0.54) a	6.04 ±(1.09) a	0.088
EIP	µg P/g-DW	2.68 ±(1.74) a	1.35 ±(0.38) a	3.95 ±(1.1) a	**0.042**
pH		5.29 ±(0.39) a	5.08 ±(0.12) a	5.55 ±(0.31) a	0.543
Soil moisture	%	68 ±(2) a	15 ±(1) b	13 ±(2) b	**$6.36e^{-09}$**

Abbreviation: EOC (extractable organic C); EON (extractable organic N); EOP (extractable organic P); EIP (extractable inorganic P)

Supplementary Table 2. Sum of PLFA biomarkers divided into groups. Numbers represent means ± standard errors. Units are expressed as nmol C gram dry soil^{-1}. At the bottom, reported p-values after ANOVA testing for effects of drought (bolded when below 0.05) and letters represent results from a two-sided Tukey's HSD post hoc test.

**Treatment**	**Total PLFA**	**Fungi**	**Gram positive**	**Gram negative**	**Actino**	**Protozoa**	**F:B ratio**	**AMF (NLFA)**
Control	8494 ±(432)a	2746 ±(231)a	1454 ±(30) a	902 ±(44)a	445 ±(41) a	115 ±(11) a	1.14 ±(0.08)a	796 ±(37) a
1-year drought	7545 ±(482)a	2542 ±(170)a	1233 ±(110)ab	745 ±(46) ab	382 ±(25) a	62 ±(2) b	1.26 ±(0.08)a	598 ±(26) a
10-years drought	7264 ±(237)a	2485 ±(94) a	1107 ±(27) b	686 ±(30) b	401 ±(42) a	72 ±(7) b	1.34 ±(0.02)a	592 ±(135)a
				ANOVA p-values				
Drought	0.126	0.559	**0.016**	**0.012**	0.481	**0.001**	0.150	0.192

Supplementary Table 3. List of potential EEAs measured. Stated nutrient cycles indicate which element (C,N,P or S) is targeted for acquisition by the respective enzyme. A full list with enzyme names, abbreviations used in this manuscript, functions and substrates used are given.

	Enzyme	Abbreviation	Enzyme function	Substrate
C-cycle	Exoglucanase (cellobiosidase)	CB†	Releases cellobioside from cellulose	4-Methylumbelliferyl β-D-cellobioside
	ß-Glucosidase	BG	Releases glucose from cellulose and other β-glucans	4-Methylumbelliferyl β-D-glucopyranoside
	ß-Xylosidase	XYL	Degrades hemicellulose	4-Methylumbelliferyl-β-D-xylopyranoside
	Lipase (Lipid esterases)	LIP	Degrades lipids	4-Methylumbelliferyl oleate
N-cycle*	Endochitinase	END†	Chitin decomposition and peptidoglycan decomposition	4-Methylumbelliferyl β-D-N,N′,N′′-triacetylchitotrioside
	Exochitinase (N-acetyl-β-glucosaminidase)	NAG	Chitin decomposition (releases glucosamine from the end of chitin)	4-Methylumbelliferyl N-acetyl-β-D-glucosaminide
	Leucine-Aminopeptidase (Protease)	LAP†	Degrades peptides/proteins	L-Leucine-7-amido-4-methylcoumarin
	Tyrosine-aminopeptidase (Protease)	TAP	Degrades peptides/proteins	L-Tyrosine 7-amido-4-methylcoumarin
P-cycle	Acid Phosphatase	PHOS†	Releases inorganic P from organic phosphorus	4-Methylumbelliferyl phosphate
S-cycle*	Sulfatase (Aryl-sulfate sulfohydrolase)	SULF	Releases sulfate from organic sulfur compounds	4-Methylumbelliferyl sulfate

*: please note that N and S-cycle enzymes are also related to C cycle, as they do not release inorganic form of N but compounds which contains both N and C.

†: these enzymes were analyzed during the 2011 and 2012 campaigns

Supplementary Table 4. List of primers used in this study.

Primer name	Target	Target group	Primer sequence (5’-3’)	Reference
515Fmod	SSU rRNA (V4)	Archaea and bacteria	GTGYCAGCMGCCGCGGTAA	Apprill et al., 2015
806Rmod	SSU rRNA (V4)	Archaea and bacteria	GGACTACNVGGGTWTCTAAT	Parada et al., 2016
ITS1Fmod	ITS1	Fungi	CTTGGTCATTTAGAGGAAGTAA	White et al., 1990<br>Smith and Peay, 2014
ITS2mod	ITS1	Fungi	GCTGCGTTCTTCATCGATGC	White et al., 1990<br>Smith and Peay, 2014
gITS7ngs	ITS2	Fungi	GTGARTCATCRARTYTTTG	Tedersoo and Lindahl, 2016
ITS4ngs	ITS2	Fungi	CCTSCSCTTANTDATATGC	Tedersoo and Lindahl, 2016
SSUmAf1<br>SSUmAf2	SSU rRNA – ITS – LSU rRNA	Arbuscular mycorrhizal fungi	TGGGTAATCTTTTGAAACTTYA<br>TGGGTAATCTTRTGAAACTTCA	Krueger et al.,2009
LSUmAr1<br>LSUmAr2<br>LSUmAr3<br>LSUmAr4	SSU rRNA – ITS – LSU rRNA	Arbuscular mycorrhizal fungi	GCTCACACTCAAATCTATCAAA<br>GCTCTAACTCAATTCTATCGAT<br>TGCTCTTACTCAAATCTATCAAA<br>GCTCTTACTCAAACCTATCGA	Krueger et al.,2009
AM5.8S_ILfor	ITS2	Arbuscular mycorrhizal fungi	TCGCATCGATGAAGAACG	Paymaneh et al., 2019
ITS4	ITS2	Arbuscular mycorrhizal fungi	TCCTCCGCTTATTGATATGC	White et al., 1990

Supplementary Table 5. Reported statistics for all the results presented in the main article, organized by figure and variables (for variable abbreviations refer to the main article). Reported statistics include: degrees of freedom (d.o.f.), F-values, p-values and adjusted *p*-values (adjusted for false discovery rates of 5%, see main article's Materials and Methods for details). Effect size estimate (η^2) and 90% confidence intervals are reported. Significant results (*p*-value < 0.05) are bolded.

Corresponding figure	Variable	d.o.f.	F-values	η^2 effect size	Confidence intervals	*p*-values	Adjusted *p*-value
Figure 1	MBC	2	2.022	0.31	[0.00, 0.59]	0.1882	0.2340
	MBN	2	5.702	0.56	[0.07, 0.76]	**0.0251**	**0.0445**
	MBP	2	4.436	0.50	[0.01, 0.72]	**0.0456**	0.0656
	C:N	2	5.720	0.56	[0.07, 0.76]	**0.0249**	**0.0445**
	C:P	2	8.060	0.64	[0.19, 0.80]	**0.0098**	**0.0252**
	N:P	2	1.762	0.28	[0.00, 0.57]	0.2261	0.2600
Figure 3	CB	2	1.539	0.25	[0.00, 0.55]	0.2662	0.2783
	BG	2	3.197	0.42	[0.00, 0.67]	0.0893	0.1209
	END	2	4.908	0.52	[0.03, 0.73]	**0.0362**	0.0555
	NAG	2	1.682	0.27	[0.00, 0.56]	0.2395	0.2623
	PHOS	2	6.534	0.59	[0.12, 0.78]	**0.0176**	**0.0369**
	LAP	2	14.58	0.76	[0.41, 0.87]	**0.0015**	**0.0058**
	TAP	2	18.79	0.81	[0.50, 0.90]	**0.0006**	**0.0058**
	XYL	2	6.685	0.60	[0.12, 0.78]	**0.0166**	**0.0369**
	LIP	2	0.819	0.15	[0.00, 0.45]	0.4714	0.4714
	SULF	2	14.75	0.77	[0.41, 0.87]	**0.0014**	**0.0058**
	C:N	2	1.964	0.31	[0.00, 0.59]	0.1933	0.2340
	C:P	2	14.82	0.77	[0.43, 0.88]	**0.0012**	**0.0058**
	N:P	2	8.668	0.65	[0.20, 0.81]	**0.0089**	**0.0252**
Figure 6	Soil functions	2	5.514	0.55	[0.08, 0.75]	**0.0273**	**0.0449**
	Archea/bacteria	1	44.969	0.88	[0.61, 0.94]	**0.0005**	**0.0058**
	Fungi	1	32.86	0.85	[0.51, 0.93]	**0.0012**	**0.0058**
	AMF	1	23.002	0.86	[0.55, 0.93]	**0.0030**	**0.0099**
