# Supplementary material for: Ecological memory of recurrent drought modifies soil processes via changes in soil microbial community
Source: Nat Commun. 2021 Sep 6;12:5308. doi: 10.1038/s41467-021-25675-4 (PMC8421443; doi:10.1038/s41467-021-25675-4)
Supplement: Supplementary file 3 — Description of Additional Supplementary Files. [file 41467_2021_25675_MOESM3_ESM.pdf]

Description of Additional Supplementary Files:

File Name: Supplementary Data 1

Description: The file includes all the data used in the main manuscript except for the amplicon sequencing data and analysis which is deposited in an external repository (see ‘Data Availability’).
